# RcdMathLib: An Open Source Software Library for Computing on Resource-Limited Devices

**DOI:** 10.3390/s21051689

**Published:** 2021-03-01

**Authors:** Zakaria Kasmi, Abdelmoumen Norrdine, Jochen Schiller, Mesut Güneş, Christoph Motzko

**Affiliations:** 1 Freie Universität Berlin, Department of Mathematics and Computer Science, Takustraße 9, 14195 Berlin, Germany; zakaria.kasmi@fu-berlin.de (Z.K.); jochen.schiller@fu-berlin.de (J.S.); 2 Technische Universität Darmstadt, Institut für Baubetrieb, El-Lissitzky-Straße 1, 64287 Darmstadt, Germany; c.motzko@baubetrieb.tu-darmstadt.de; 3 Otto-von-Guericke University, Faculty of Computer Science, Universitätsplatz 2, 39106 Magdeburg, Germany; mesut.guenes@ovgu.de

**Keywords:** singular value decomposition, trilateration, Gauss–Newton, Levenberg–Marquardt, multipath recognition and mitigation, positioning, RIOT-OS, microcontrollers, embedded systems, internet of things

## Abstract

We developped an open source library called RcdMathLib for solving multivariate linear and nonlinear systems. RcdMathLib supports on-the-fly computing on low-cost and resource-constrained devices, e.g., microcontrollers. The decentralized processing is a step towards ubiquitous computing enabling the implementation of Internet of Things (IoT) applications. RcdMathLib is modular- and layer-based, whereby different modules allow for algebraic operations such as vector and matrix operations or decompositions. RcdMathLib also comprises a utilities-module providing sorting and filtering algorithms as well as methods generating random variables. It enables solving linear and nonlinear equations based on efficient decomposition approaches such as the Singular Value Decomposition (SVD) algorithm. The open source library also provides optimization methods such as Gauss–Newton and Levenberg–Marquardt algorithms for solving problems of regression smoothing and curve fitting. Furthermore, a positioning module permits computing positions of IoT devices using algorithms for instance trilateration. This module also enables the optimization of the position by performing a method to reduce multipath errors on the mobile device. The library is implemented and tested on resource-limited IoT as well as on full-fledged operating systems. The open source software library is hosted on a GitLab repository.

## 1. Introduction

Algorithms and scientific computing are workhorses of many numerical libraries that support users to solve technical and scientific problems. These libraries use mathematics and numerical algebraic computations, which contribute to a growing body of research in engineering and computational science. This leads to new disciplines and academic interests. The use of computers has accelerated the trend as well as enhanced the deployment of numerical libraries and approaches in scientific and engineering communities. Originally, computers were built for numerical and scientific applications. Konrad Zuse built a mechanical computer in 1938 to perform repetitive and cumbersome calculations. A specific problem, from the area of static engineering, requires performing tedious calculations to design load-bearing structures by solving systems of linear equations [1]. Howard Aiken independently developed an electro-mechanical computing device that can execute predetermined commands typed on a keyboard in the notation of mathematics and translated into numerical codes. These are stored on punched cards and perforated magnetic tapes or drums [2,3].

The first software libraries of numerical algorithms were developed in the programming language ALGOL 60 including procedures for solving linear systems of equations or eigenvalue problems [4]. This software was rewritten in FORTRAN and ported to the LINPACK and EISPACK software packages [5,6]. Cleve Moler implemented a user-friendly interface to enable his students an easy access to LINPACK and EISPACK without writing Fortran programs [7]. He called the interface MATLAB (Matrix Laboratory), which was so successful that he founded a company called MathWorks. MATLAB is now a full-featured computing platform.

Ubiquitous computing on resource-limited devices has become an important issue in the Internet of Things (IoT) and the Machine to Machine (M2M) communication technologies, enabling the implementation of various applications such as health monitoring or vehicle tracking and detection. IoT is an emerging and challenging technology that facilitates the realization of computing services in various areas by using advanced communication protocols, technologies, and intelligent data analytical software [8]. The M2M communication in combination with the Radio-Frequency Identification (RFID), localization, observation by sensors, and controlling of actuators provide context-aware intelligent decisions as well as high-quality services. Computing plays a key role in implementing such applications, particularly by applying local and decentral processing on mobile and ubiquitous devices. This affords in-network and local context-aware decisions without the use of external computing services (e.g., cloud services). Therefore, we provide an open source software library for numerical linear algebra called RcdMathLib (Mathematical Library for Resource-constrained devices) [9]. This software library is suitable for devices with limited resources such as microcontrollers or portable computing devices. These devices are mostly low-cost, are equipped with low-end processors, and have limited memory and energy resources. RcdMathLib supports a decentralized and on-the-fly numerical computing locally on a mobile device. The decentralized numerical calculations allow for pushing the application-level knowledge into the mobile device and avoiding the communication as well as the calculation on a central unit such as a processing server. The decentralization allows for the reduction of latency and processing time as well as enhancing the real-time capability because the length of the path to be traveled from data are shortened. Furthermore, RcdMathLib provides useful algorithms such as the Singular Value Decomposition (SVD) which has become an indispensable tool in science and engineering [10]. SVD is applied for image compression and restoration or biomedical applications, for example, noise reduction of biomedical signals [11].

RcdMathLib allows for computing on mobile devices as well as embedded systems providing algorithms for the solution of linear and nonlinear multivariate system of equations. The solution of these equation systems is achieved by using robust matrix decomposition algorithms. The software library offers an optimization module for curve fitting or solving of problems of regression smoothing. Applications can be built and organized as modules using the RcdMathLib, therefore, we offer a localization module. This is an application module for distance- and Direct-Current (DC)-pulsed, magnetic-based localization systems. The localization module allows for a position estimation of a mobile device. Localization enables the realization of context-aware computing applications in combination with mobile devices. In this sense, the software library enables the computation of the localization on mobile systems. RcdMathLib also enables the optimization of the estimated location by using an adaptive approach based on the SVD, Levenberg–Marquardt (LVM) algorithms, and the Position Dilution of Precision (PDOP) value [12,13,14]. In addition, the localization module provides an algorithm for the multipath detection and mitigation enables an accurate localization of the mobile device in Non-Line-of-Sight (NLoS) scenarios. RcdMathLib can be also used on a full-fledged device such as a Personal Computer (PC) or a computing server.

In this article, we will present the RcdMathLib as well as briefly address the difficulties by using linear algebra methods and the techniques to overcome the limitation of resource-constrained devices. Our main contributions are:

- An open source library for numerical computations on resource-limited devices and embedded systems. The software permits a user or a mobile device to solve multivariant linear equation systems based on efficient algorithms such as the Householder or the Moore–Penrose inverse. The Moore–Penrose inverse is implemented by using the SVD method.
- A module for solving multivariant nonlinear equation systems as well as optimization and curve fitting problems on a resource-contained device on the basis of the SVD algorithm.
- A utilities-module provides various algorithms such as the Shell sort algorithm or the Box–Muller method to generate normally distributed random variables.
- A localization module for positioning systems that use distance measurements or DC-pulsed, magnetic signals. This module enables an adaptive, optimized localization of mobile devices.
- A software routine to locally reduce multipath errors on mobile devices.

Guckenheimer perceived that we are conducting increasingly complex computations built upon the assumption that the underlying numerical approaches are complete and reliable. Furthermore, we ignore numerical analysis by using mathematical software packages [15]. Thus, the user must be aware of the limitations of the algorithms and be able to choose appropriate numerical methods. The use of inappropriate methods can lead to incorrect results. Therefore, we briefly address certain difficulties by using linear algebra algorithms.

The remainder of this article is structured as follows: Firstly, we review related works in Section 2. We present the architecture as well as describe the modules of the software library in Section 3. We introduce the implementation issues in Section 4 and the usage of the RcdMathLib in Section 5. In Section 6, we evaluate the algorithms on a resource-limited device and on a low-cost, single-board computer. Finally, we conclude our article and give an outlook on future works in Section 7.

## 2. Related Work

Computing software especially for linear algebra is indispensable in science and engineering for the implementation of applications like text classification or speech recognition. To the best of our knowledge, there are few mathematical libraries for resource-limited devices, and most of them are limited to simple algebraic operations.

Libraries for numerical computation such as the GNU Scientific Library (GSL) are suitable for Digital Signal Processors (DSPs) or Linux-based embedded systems. For example, the commercially available Arm Performance Libraries (ARMPL) offer basic linear algebra subprograms, fast Fourier transform routines for real and complex data as well as some mathematical routines such as exponential, power, and logarithmic routines. Nonetheless, these routines do not support resource-constrained devices such as microcontrollers [16].

The C standard mathematical library includes mathematical functions defined in <math.h>. This mathematical library is widely used for microcontrollers, since it is a part of the C compiler. It provides only basic mathematical functions for instance trigonometric functions (e.g., sin, cos) or exponentiation and logarithmic functions (e.g., exp, log) [17].

Our research shows that there are very few attempts to build numerical computations libraries, which can run on microcontrollers: Texas Instruments^®^ (Dallas, Texas, USA) provides for its MSP430 and MSP432 devices the IQmath and Qmath Libraries, which contain a collection of mathematical routines for C programmers. However, this collection is restricted only to basic mathematical functions such as trigonometric and algorithmic functions [18].

Libfixmatrix is a matrix computation library for microcontrollers. This library includes basic matrix operations such as multiplication, addition, and transposition. Equation solving and matrix inversion are implemented by the QR decomposition. Libfixmatrix is only suitable for tasks involving small matrices [19].

MicroBLAS is a simple, tiny, and efficient library designed for PC and microcontrollers. It provides basic linear algebra subprograms on vectors and matrices [20].

Other libraries for microcontrollers are the MatrixMath and BasicLinearAlgebra libraries. However, these libraries offer limited functionalities and are restricted to the Arduino platform [21,22].

The Python programming language is becoming widely used in scientific and numeric computing. The Python package NumPy (Numeric Python) is used for manipulating large arrays and matrices of numeric data [23]. The Scientific Python (SciPy) extends the functionality of NumPy with numerous mathematical algorithms [24]. Python is widely used for PC and single-board computers such as the Raspberry Pi. A new programming language largely compatible with Python called MicroPython is optimized to run on microcontrollers [25]. MicroPython includes the math and cmath libraries, which are restricted to basic mathematical functions. The mainline kernel of MicroPython supports only the ARM Cortex-M processor family. CircuitPython is a derivative of the MicroPython created to support boards like the Gemma M0 [26]. Various mathematical libraries are outlined in Table 1, which reveals their capabilities, limitations, and supported platforms.

## 3. Library Architecture and Description

**Figure 1:**
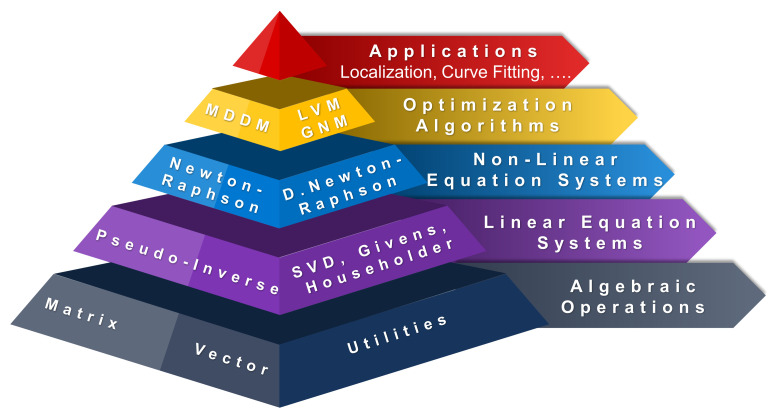
Architecture of the RcdMathLib. SVD, Singular Value Decomposition. D. Newton-Raphson, Damped Newton-Raphson. LVM, Levenberg–Marquardt Method. GNM, Gauss–Newton Method. MDDM, Multipath Distance Detection and Mitigation.

RcdMathLib has a pyramidal and a modular architecture as illustrated in Figure 1, whereby each layer rests upon the underlying layers. For example, the linear algebra module layer rests on the basic algebraic module layer. Each module layer is composed of several submodules such as the matrix or vector submodules. The submodules can be built up from the underlying submodules, for example, the pseudo-inverse submodule is based on the SVD, the Householder, and the Gevins submodules. For the sake of brevity, Figure 1 presents only the sublayers. The software layers will be briefly addressed in Section 3.1 through Section 3.3.

### 3.1. Linear Algebra Module Layer

The module layer of linear algebra is composed of the following submodules:

- Basic operations submodule: provides algebraic operations such as addition or multiplication of vectors or matrices. This submodule distinguishes between vector and matrix operations.
- Matrix decomposition submodule: allows for the decomposition of matrices by using algorithms such as Givens, Householder, or the SVD. The SVD method is implemented using the Golub–Kahan–Reinsch algorithm [27,28].
- Pseudo-inverse submodule: enables the computation of the inverse of quadratic as well as of rectangular matrices. The matrix inverse can be calculated by using the Moore–Penrose, Givens, or Householder algorithms [29].
- Linear solve submodule: permits the solution of under-determined and over-determined linear equation systems. We solve the linear equation systems using two matrix decompositions: the SVD and QR factorizations. The first method uses the Moore–Penrose inverse, while the second approach applies the Householder or the Givens algorithms with the combination of the back substitution method. We also provide the Gaussian Elimination (GE) with a pivoting algorithm, which is based on the LU decomposition. We use the GE-based method only for testing purposes or for devices with very limited stack memory. We suggest using the SVD- or the QR-based methods due to the numerical stability and the support of non-quadratic matrices [30].
- Utilities submodule: offers filtering algorithms such as median, average, or moving average. Furthermore, it provides the Shell algorithm to put elements of a vector in a certain order as well as the Box–Muller method to generate normally distributed random variables [31,32].

### 3.2. Non-Linear Algebra Module Layer

The nonlinear algebra module includes the following submodules:

- Optimization submodule: enables the optimization of an approximate solution by using Nonlinear Least Squares (NLS) methods such as modified Gauss–Newton (GN) or the LVM algorithms. These methods are iterative and need a start value as an approximate solution. Moreover, the user should give a pointer to an error function and to a Jacobian matrix. The modified GN and the LVM algorithms will be briefly described in Section 3.2.1 and Section 3.2.2.
- Nonlinear equations submodule: allows for the solution of multivariate nonlinear equation systems by using Newton–Raphson and damped Newton–Raphson methods [33]. The user must deliver a start value as well as a pointer to nonlinear equation systems to solve, and a pointer to the appropriate Jacobian matrix.

#### 3.2.1. Gauss–Newton Algorithm

The Gauss–Newton algorithm works iteratively to find the solution $\vec{x}$ that minimizes the sum of the square errors. During the iteration process, we cache the value of $\vec{x}$ with the minimal sum of squares to prevent the divergence of the GN algorithm [34]. The computed solution by the GN can be used as a start value for the subsequent LVM algorithm if the start value is unknown or the GN algorithm diverges.

#### 3.2.2. Levenberg–Marquardt Algorithm

The LVM algorithm is also a numerical optimization approach enabling solving NLS problems [35,36,37]. The LVM algorithm can be used for optimization or fitting problems. The LVM method proceeds iteratively as follows:

(1) $${\vec{x}}^{\;(k+1)}={\vec{x}}^{\;(k)}+{\vec{s}}^{\;(k)},$$

where ${\vec{x}}^{\;(k)}$ is the *k*-th approximation of the searched solution and ${\vec{s}}^{\;(k)}$ is the *k*-th error correction vector. The LVM improves the approximate solution $\vec{x_{0}}$ in each iteration step by calculating the correction vector ${\vec{s}}^{\;(i)}$ as follows [38,39]:

(2) $$\left(J_{f}^{T}\left({\vec{x}}^{\;(i)}\right)J_{f}\left({\vec{x}}^{\;(i)}\right)+{\left({µ}^{\left(i\right)}\right)}^{2}I\right){\vec{s}}^{\;(i)}=-J_{f}^{T}\left({\vec{x}}^{\;(i)}\right)\vec{f}\left({\vec{x}}^{\;(i)}\right),$$

where µ is the damping parameter, *f* is the error function, and $J_{f}$ is the Jacobian matrix. The LVM algorithm has the advantage over the GN method because the matrix on the left side of Equation (2) is no longer singular. This is accomplished by the factor ${µ}^{2}I$ regulating the matrix $J_{f}^{T}J_{f}$. The LVM method is described in Algorithm 1.
**Algorithm 1** LVM algorithm1:**function**LVM_ALG($\varepsilon_{x},\beta_{0},\beta_{1},\tau ,i_{max},{\vec{x}}^{\;(0)},\vec{f},J_{f}$)2:  i=0; $\vec{x}={\vec{x}}^{\;(0)}$; $B=J_{f}^{T}\left(\vec{x}\right)J_{f}\left(\vec{x}\right)$; $\vec{H}=J_{f}^{T}\left(\vec{x}\right)\vec{f}\left(\vec{x}\right)$;3:  ${µ}^{\left(0\right)}=\tau \cdot \max_{i}\left\{b_{ii}\left(\vec{x}\right)\right\}$; $µ={µ}^{\left(0\right)}$;4:  Solve $\left(B+{µ}^{2}I\right)\vec{s}=-\vec{H}$ for $\vec{s}$;5:  **while** $((∥\vec{s}{∥}_{2}>\varepsilon_{x}(1+∥\vec{x}{∥}_{2})\;\mathrm{and}\;\left(i<i_{max}\right))$ **do**6:   $\left[\vec{s},µ\right]$ = CORRECTION_FUNC($\vec{x},µ,\beta_{0},\beta_{1}$);7:   **while** (true) **do**8:    **if** $\left(\rho_{µ}\leq \beta_{0}\right)$ **then**9:     µ=2µ10:     $\left[\vec{s},\rho_{µ}\right]$ = CORRECTION_FUNC($µ,\beta_{0},\beta_{1}\vec{x},\vec{f},J_{f}$);11:    **else if** $\left(\rho_{µ}\geq \beta_{1}\right)$ **then**12:     $µ=\frac{µ}{2}$13:     break;14:    **else**15:     break;16:    **end if**17:   **end while**18:   $\vec{x}=\vec{x}+\vec{s}$;19:   i=i+1;20:  **end while**21:**end function**1:**function**correction_func($µ,\beta_{0},\beta_{1}\vec{x},\vec{f},J_{f}$)2:  $B=J_{f}^{T}\left(\vec{x}\right)J_{f}\left(\vec{x}\right)$; $\vec{H}=J_{f}^{T}\left(\vec{x}\right)\vec{f}\left(\vec{x}\right)$;3:  Solve $\left(B+{µ}^{2}I\right)\vec{s}=-\vec{H}$ for $\vec{s}$;4:  $\rho_{µ}=\frac{∥\vec{f}\left(\vec{x}\right){∥}_{2}^{2}-{∥\vec{f}\left(\vec{x}+\vec{s}\right)∥}_{2}^{2}}{∥\vec{f}\left(\vec{x}\right){∥}_{2}^{2}-∥\vec{f}\left(\vec{x}\right)+J_{f}\left(\vec{x}\right)\vec{s}{)∥}_{2}^{2}}$;5:**end function**

### 3.3. Localization Module Layer

Localization of users or IoT devices is indispensable for Localization-Based Services (LBSs) such as tracking in smart buildings, advertising in shopping centers, or routing and navigation in large public buildings [40]. Indoor Localization Systems (ILSs) are used to locate IoT or mobile devices inside environments where the Global Positioning System (GPS) cannot be deployed. Numerous technologies have been evaluated for ILSs, for example, Ultra-Wideband (UWB) [41], Wireless Local Area Network (WLAN) [42], ultrasound [43], magnetic signals [44], or Bluetooth [45].

#### 3.3.1. Introduction and Layer Description

The Localization Module (LM) layer provides algorithms to calculate as well as optimize a position of an ILS. At the current stage of development, we provide two example applications of the LM as submodules: a distance-based as well as a DC-pulsed, magnetic-based submodule of ILSs. We also offer a common positioning submodule comprising shared algorithms like the trilateration algorithm [46].

**Figure 2:**
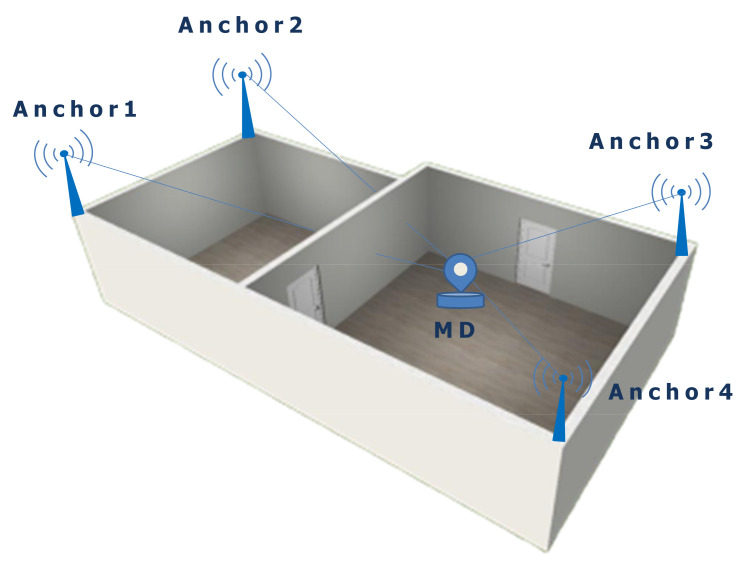
Distance-based Indoor Localization System. MD, Mobile Device.

Figure 2 illustrates the principle of a distance-based ILS composed of four anchors with known positions and one mobile device. The distances between the anchors and the mobile device can be measured by using UWB or ultrasound sensors. The mobile device performs distance measurements to the four anchors. Furthermore, the collected distances are preprocessed using the median filter from the utilities submodule to remove outliers (see Section 3.1). Finally, the mobile device locally calculates a three-dimensional position using the trilateration algorithm provided by the RcdMathLib.

The trilateration algorithm computes the position of an unknown point with the coordinates (x,y,z) and distances $d_{i}$ to the reference positions $\left(x_{i},y_{i},z_{i}\right)$ for i=1,2,…,n. This problem requires the estimation of a vector $\vec{x}=\left(w,x,y,z\right)$ such that:

(3) $$A\vec{x}=\vec{b},$$

where the matrix A and the vector $\vec{b}$ have the following forms [46,47,48]:

(4) $$A=\left[\begin{array}{cccc} 1 & -2x_{1} & -2y_{1} & -2z_{1}\\ 1 & -2x_{2} & -2y_{2} & -2z_{2}\\ 1 & -2x_{3} & -2y_{3} & -2z_{3}\\ \vdots & \vdots & \vdots & \vdots \\ 1 & -2x_{n} & -2y_{n} & -2z_{n} \end{array}\right]\;\mathrm{and}\;$$

(5) $$\vec{b}=\left[\begin{array}{c} d_{1}^{2}-x_{1}^{2}-y_{1}^{2}-z_{1}^{2}\\ d_{2}^{2}-x_{2}^{2}-y_{2}^{2}-z_{2}^{2}\\ d_{3}^{2}-x_{3}^{2}-y_{3}^{2}-z_{3}^{2}\\ \vdots \\ d_{n}^{2}-x_{n}^{2}-y_{n}^{2}-z_{n}^{2} \end{array}\right].$$

The solution $\vec{x}$ is given by:

(6) $$\vec{x}=A^{+}\vec{b},$$

where $A^{+}$ is the pseudo-inverse of the matrix *A*. The pseudo-inverse matrix $A^{+}$ is computed by using the pseudo-inverse submodule of the RcdMathLib (see Section 3.1). The quality *q* of the calculated position $\vec{x}$ is given by:

(7) $$q=w-(x^{2}+y^{2}+z^{2}).$$

**Figure 3:**
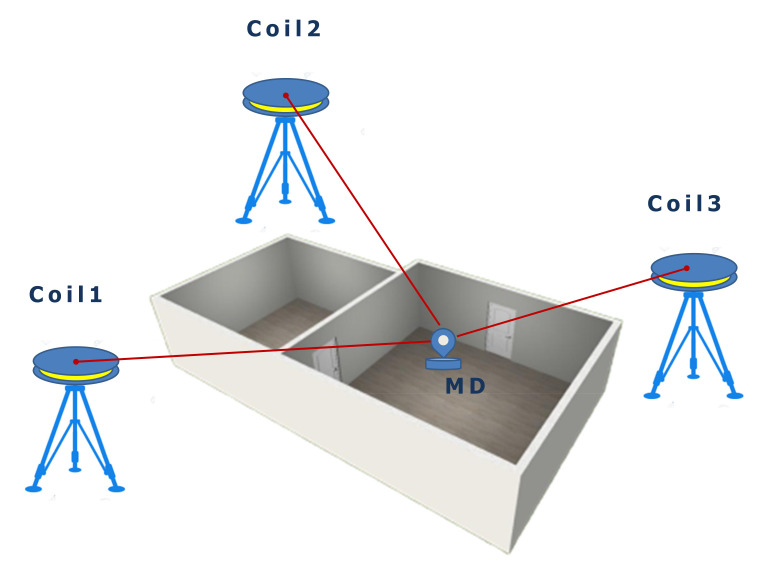
Magnetic-based indoor localization system. MD, Mobile Device.

Figure 3 illustrates the principle of a magnetic-based ILS composed of various coils with known positions and a mobile device. This system enables the calculation of the position of the mobile device by measuring magnetic fields to the coils as anchors. The magnetic signals are artificially generated from the coils using a pulsed direct current. The collected measurement data are preprocessed from the utilities-submodule for removing outliers and calibrating magnetic data. Finally, the position is calculated on the mobile device.

The magnetic field $B_{i}$ generated from the coil *i* is equal to [47,49]:

(8) $$\begin{array}{cc} B_{i} & =\frac{K}{r_{i}^{3}}\sqrt{1+3\sin^{2}\left(\theta_{i}\right)}\;\;\;i=1,2,\ldots ,n. \end{array}$$

In this setting, $K=\frac{{µ}_{0}N_{t}IF}{4\pi }$, where $N_{t}$ describes the number of turns of the wire, *I* is the current running through the coil, *F* expresses the base area of the coil, ${µ}_{0}$ is the permeability of free space, $r_{i}$ is the distance between the mobile device and coil *i*, and $\theta_{i}$ is the mobile device elevation angle relative to the coil plane. The distance $r_{i}$ and the elevation angle $\theta_{i}$ are equal to:

(9) $$\begin{array}{cc} r_{i} & =\sqrt{{\left(x-x_{i}\right)}^{2}+{\left(y-y_{i}\right)}^{2}+{\left(z-z_{i}\right)}^{2}} \end{array}$$

(10) $$\begin{array}{cc} \sin\theta_{i} & =\frac{z-z_{i}}{r_{i}}. \end{array}$$

Equation (8) is a nonlinear equation system with the unknowns coordinates *x*, *y* and *z*, which can be solved by applying the LVM algorithm from the optimization-submodule of the RcdMathLib, whereby, $\left(x_{i},y_{i},z_{i}\right)$ and (x,y,z) are the coordinates of the *i*-th coil and the mobile device.

#### 3.3.2. Multipath Distance Detection and Mitigation and Position Optimization Algorithm

The location module is not restricted to simple position calculations but rather performs complex tasks such as the Multipath Distance Detection and Mitigation (MDDM) by the usage of other modules of the RcdMathLib. The MDDM algorithm enables to reduce the effects of multipath fading on digital signals in radio environments of a mobile device to Reference Stations (RSs) with known locations. The MDDM approach is based on the Robust Position Estimation in Ultrasound Systems (RoPEUS) algorithm [50]. The MDDM is adapted for precise distance-based ILSs such as the Ultra-Wideband (UWB)-based localization systems, whereas it is simultaneously optimized for resource-limited devices [12]. The MDDM algorithm is summarized in Algorithm 2.
**Algorithm 2** MDDM algorithm
1: **function**
recog_mitigate_multipath_alg(k,n,threshold,d,RS)
2:    j=0; $r_{min}=\infty$;
3:    distances $d_{i}$, i=0,…,n−1;▹ distance measures to *n* RSs4:    **while**
(j<nk)
**do**
5:        $comb\left(k\right)=RS_{0}\ldots RS_{k-1}$;▹ choose *k* RSs6:        $\vec{x_{k}}$▹ Compute a position related to *k* RSs7:        $r_{i}=R_{i}-d_{i}$;▹ residuals8:        $r=\sum_{i=0}^{n-1}r_{i}$;
9:        $r_{min}=min\left(r,r_{min}\right)$;
10:        j=j+1;
11:    **end while**
12:    ${\vec{x}}_{0}=\underset{r_{min}}{arg min}$▹ the solution13:    $PDOP_{x}$▹ calculate PDOP-value of $\vec{x}$14:    **if**
$\left(PDOP_{x}>threshold\right)$
**then**
15:        LVM_ALG($\varepsilon_{x},\beta_{0},\beta_{1},\tau ,i_{max},{\vec{x}}_{0},\vec{f},J_{f}$)▹ optimize the position16:    **end if**
17: **end function**


### 3.4. Documentation and Examples’ Modules

RcdMathLib includes a module that provides an Application Programming Interface (API) documentation. The API documentation is in Portable Document Format (PDF) and in Hypertext Markup Language (HTML) format. It is generated from the C source code by using the Doxygen tool [51]. The software reference documentation covers the description of the implemented functions as well as their passing parameters. In addition, the example module comprises samples of each module to familiarize the users with the API. The example module has the same structure as the RcdMathLib.

## 4. Implementation Issues

Given a (m×n) non-singular matrix *A* and an *n*-vector $\vec{b}$, the fundamental problem of linear algebra is to find an *n*-vector $\vec{x}$ such that $A\vec{x}=\vec{b}$. This fundamental problem emerges in various areas of science and engineering such as applied mathematics, physics, or electrical engineering [52]. Associated problems are finding the inverse, the rank, or projections of a matrix *A*. Attempting to solve the linear algebra problem using common theoretical approaches would face computational difficulties. For example, solving a (20,20) linear system with the Cramer’s Rule, which is a significant theoretical algorithm, might take more than a million years even by using fast computers [52].

We use the Givens, the Householder, and the SVD matrix decomposition algorithms. These decomposition methods enable the solution of various problems such as the computation of the inverse matrix, the linear equations, or the rank of a matrix. We do not use the Cholesky decomposition ($AA^{T}$), since it can become unstable due to the rounding errors that are equal to ${\left[\kappa \left(A\right)\right]}^{2}$ instead of $\kappa \left(A\right)$. Although the Gaussian Elimination (GE) is an efficient algorithm to implement the LU factorization, we do not used it, since GE generally requires pivoting and is limited to square matrices. Furthermore, GE can be unstable in certain contrived cases; nonetheless, it performs well for the majority of practical problems [53,54].

We do not use the Classical Gram–Schmidt (CGS) and the Modified Gram–Schmidt (MGS) to implement the QR decomposition due to the numerical instability. Instead, we use the Householder and the Givens algorithms. Even though the Householder is more efficient than the Givens algorithm, the Givens method is easy to parallelize. The Givens and the Householder methods have a guaranteed stability but fail if the matrix is nearly rank-deficient or singular. The SVD algorithm can be used to avoid the rank deficiency problem. This algorithm is not be explicitly computable by determining the eigenvalues of the symmetric matrix $A^{T}A$ due to the round-off errors in the calculation of the matrix $A^{T}A$. Therefore, we implement the SVD by using the Golub–Kahah–Reinsch (GKR) algorithm, which will be described in Section 4.1.

We calculate the pseudo-inverse matrix by using the Moore–Penrose method based on the SVD or the QR decomposition using the Householder or the Givens algorithms. The QR-based pseudo-inverse $A^{+}$ is computed as follows:

(11) $$\begin{array}{cc} A & =QR, \end{array}$$

(12) $$\begin{array}{cc} A^{+} & =A^{-1}={\left(QR\right)}^{-1}=R^{-1}Q^{-1}, \end{array}$$

(13) $$\begin{array}{cc} A^{+} & =R^{-1}Q^{T}, \end{array}$$

where $R^{-1}$ is the inverse of an upper triangular matrix, and $Q^{T}$ is the transpose of an orthogonal matrix. We calculate the $R^{-1}$ matrix by using Algorithm 3 [55].
**Algorithm 3** Inverse of an upper triangular matrix1:**function**inv_upper_triang_matrix_alg(n,U)2:    $U_{inv}$;                   ▹ holds the calculated inverse of the matrix *U*3:    **for** i=0**to**n−1 **do**4:        $U_{inv}\left[i,i\right]=1/U\left[i,i\right]$;5:        **for** j=0**to**i−2 **do**6:           $U_{inv}\left[j,i\right]=-U\left[i,i\right]\left(U[j,j:i-2]U[j:i-2,i]\right)$;7:        **end for**8:    **end for**9:**end function**

In general, we avoid the explicit calculation of matrix multiplications such as the construction of Householder matrices ($H_{i}A$) or of Givens matrices ($J_{i}A$). The calculated triangular matrix *R* is stored over the matrix *A*. We also provide functions to avoid the explicit computation and storage of the transpose matrix such as the function that implicitly calculates the matrix–transpose–vector multiplication ($A^{T}\vec{x}$). We use the SVD algorithm to overcome the rank deficiency problem, for example, by the modified GN, the Newton–Raphson, and damped Newton–Raphson methods. We used the Householder instead of the SVD method by the LVM algorithm to save computing time and memory stack. This optimization is possible because of the robustness of the LVM algorithm (see Section 3.2.2). We provide the iterative Shell sort algorithm that is suitable for resource-limited devices with a limited stack size. We use the Shell sort algorithm to implement the median filter.

### 4.1. Singular Value Decomposition

The SVD method has become a powerful tool for solving a wide range of problems in different application domains such as biomedical engineering, control systems, or signal processing [52]. We implemented the SVD approach based on the Golub–Kahan–Reinsch algorithm that works in two phases: a bidiagonalization of the matrix *A* and the reduction of the calculated bidiagonal matrix to a diagonal form.

**First phase (bidiagonalization)** 
  A (m×n) matrix *A* is transformed to an upper bidiagonal matrix $B\in R^{mxn}$ by using the Householder bidiagonalization, where m≥n. The matrix *A* is transformed as follows:
  (14) $$U_{0}^{T}AV_{0}^{T}=\left[\begin{array}{c} B\\ 0 \end{array}\right],$$
  where *B* is an n×n bidiagonal matrix equal to
  (15) $$\left[\begin{array}{cccc} b_{11} & b_{12} & \ldots & 0\\ 0 & \ddots & \ddots & \vdots \\ \vdots & \ldots & \ddots & b_{n-1,n}\\ 0 & 0 & 0 & b_{n,n} \end{array}\right].$$
**Second phase (reduction to the diagonal form)** 
  The bidiagonal matrix *B* is further reduced to a diagonal matrix Σ by using orthogonal equivalence transformations as follows:
  (16) $$U_{1}^{T}BV_{1}^{T}=\Sigma =\mathrm{diag}\left(\sigma_{1},\sigma_{2},\cdots ,\sigma_{n}\right),$$
  where Σ is the matrix of the singular values $\sigma_{i}$ and the matrices $U_{1}$ and $V_{1}$ are orthogonal. The singular vector matrices can be computed as follows:
  (17) $$\begin{array}{cc} U & =U_{0}U_{1}, \end{array}$$
  (18) $$\begin{array}{cc} V & =V_{0}V_{1}. \end{array}$$
  We implemented the first and second phases by the Golub–Kahan bidiagonal procedure and the Golub–Reinsch algorithm. Both algorithms will be described in detail in Section 4.1.1 and Section 4.1.2. In this description, we will mention some implementation issues.

#### 4.1.1. Golub–Kahan Bi-Diagonal Procedure

The reduction of matrix *A* to the upper bi-diagonal matrix *B* is accomplished by using a sequence of Householder reflections, where the matrix *B* has the same set of singular values as the matrix *A* [56]. First, a Householder transformation $U_{01}$ is applied to zero out the sub-diagonal elements of the first column of the matrix *A*. Next, a Householder transformation $V_{01}$ is used to zero out the last (n−2) elements of the first row by post-multiplying the matrix $U_{01}A$: $U_{01}AV_{01}$. Repeating these steps a total of *n* times, the matrix *A* will be transformed to:

(19) $$B=\left(U_{n}U_{n-1}\ldots U_{1}U_{0}\right)A\left(V_{n}V_{n-1}\ldots V_{1}V_{0}\right).$$

#### 4.1.2. Golub–Reinsch Algorithm

The Golub–Reinsch algorithm is a variant of the QR iteration [28]. At each iteration *i*, the implicit symmetric QR algorithm is applied with the Wilkinson shift without forming the product $B_{i}^{T}B_{i}$. The algorithm has guaranteed convergence with a quite fast rate [52]. Starting from the bi-diagonalization of the matrix *A* obtained from the previous Golub–Kahan bi-diagonal procedure, the algorithm creates a sequence of bi-diagonal matrices $\left\{B_{i}\right\}$ with possibly smaller off-diagonals than the previous one. For simplicity, we write:

(20) $$B=\left[\begin{array}{cccc} \alpha_{1} & \beta_{2} & \; & \\ \; & \ddots & \ddots & \\ \; & \; & \ddots & \beta_{n}\\ \; & \; & & \alpha_{n} \end{array}\right]$$

We calculate the Wilkinson shift σ that is equal to the eigenvalue λ of the right-hand corner sub-matrix of the matrix $C=B_{i}^{T}B_{i}$:

(21) $$\left[\begin{array}{cc} c_{n-1,n-1} & c_{n-1,n}\\ c_{n-1,n} & c_{n,n} \end{array}\right]=\left[\begin{array}{cc} \alpha_{n-1}^{2}+\beta_{n-1}^{2} & \alpha_{n-1}\beta_{n}\\ \alpha_{n-1}\beta_{n} & \alpha_{n}^{2}+\beta_{n}^{2} \end{array}\right],$$

which is closer to $\alpha_{n}^{2}+\beta_{n}^{2}$. G. H. Golub and C. F. Van Loan suggest to calculate the Wilkinson shift as follows [28]:

(22) $$\begin{array}{cc} \delta & =\frac{c_{n-1,n-1}-c_{n,n}}{2}, \end{array}$$

(23) $$\begin{array}{cc} \sigma & =c_{n,n}-\frac{\mathrm{sign}\left(\delta \right)c_{n-1,n}^{2}}{\left|\delta \right|+\sqrt{\delta^{2}+c_{n-1,n}^{2}}}. \end{array}$$

We calculate $c_{1}$ and $s_{1}$ such that:

(24) $${\left[\begin{array}{cc} c_{1} & s_{1}\\ -s_{1} & c_{1} \end{array}\right]}^{T}\left[\begin{array}{c} \alpha_{1}^{2}-\sigma \\ \alpha_{1}\beta_{2} \end{array}\right]=\left[\begin{array}{c} {}*\\ 0 \end{array}\right],$$

and form the Givens rotation $V_{1}$.

We apply the Givens rotation $V_{1}$ to the right of the matrix *B*:

(25) $$BV_{1}=\left[\begin{array}{cccc} {}* & * & \; & \\ \; & * & * & \\ \; & \; & * & *\\ \; & \; & & * \end{array}\right]V_{1}=\left[\begin{array}{cccc} {}* & * & \; & \\ + & * & * & \\ \; & \; & * & *\\ \; & \; & & * \end{array}\right].$$

The bidiagonal form is destroyed by the unwanted non-zero element (bulge) indicated by the “+” sign. Therefore, we apply the Givens rotations $U_{1}$, $V_{2}$, $U_{2}$, …, $V_{n-1}$, and $U_{n-1}$ to chase the badges.

We apply a Givens transformation $U_{1}$ to the left of the matrix $BV_{1}$ to eliminate the unwanted sub-diagonal element. This reintroduces a badge in the first row to the right of the super-diagonal element:

(26) $$U_{1}BV_{1}=\left[\begin{array}{cccc} {}* & * & + & \\ 0 & * & * & \\ \; & \; & * & *\\ \; & \; & & * \end{array}\right].$$

We apply the Givens rotations $V_{2}$ to remove the badge in the matrix $U_{1}BV_{1}$. This introduces a new badge into the sub-diagonal of the third row, which is eliminated by the Givens rotation $U_{2}$:

(27) $$U_{2}U_{1}BV_{1}V_{2}=\left[\begin{array}{cccc} {}* & * & 0 & \\ \; & * & * & \\ \; & + & * & *\\ \; & \; & & * \end{array}\right]=U_{2}\left[\begin{array}{cccc} {}* & * & \; & \\ \; & * & * & +\\ \; & 0 & * & *\\ \; & \; & & * \end{array}\right].$$

The matrix pair ($V_{3}$, $U_{3}$) terminates the chasing process and delivers a new bi-diagonal matrix $\tilde{B}$:

(28) $$\tilde{B}=U_{3}U_{2}U_{1}BV_{1}V_{2}V_{3}.$$

In general, the chasing process creates a new bi-diagonal matrix $\tilde{B}$ that is related to the matrix *B* as follows [28]:

(29) $$\tilde{B}=\left(U_{n-1}\ldots U_{1}\right)B\left(V_{1}V_{2}\ldots V_{n-1}\right)=\tilde{U}B\tilde{V},$$

where $\tilde{U}$ and $\tilde{V}$ are orthogonal. During the chasing process, we distinguish between the splitting, the cancellation, and the negligibility steps [57]:

At the *i*-th iteration, we assume that the matrix $\tilde{B}$ is equal to:

(30) $$\tilde{B_{i}}=\left[\begin{array}{cccc} q_{1} & e_{2} & \; & \\ \; & \ddots & \ddots & \\ \; & \; & q_{n-1} & e_{n}\\ \; & \; & & q_{n} \end{array}\right].$$

**Splitting:**  If the matrix entry $e_{i}$ is equal to zero, we split the matrix $\tilde{B_{i}}$ into two block diagonal-matrices whose singular values can be computed independently:
  (31) $$\begin{array}{cc} \tilde{B_{i}} & =\left[\begin{array}{cc} \tilde{B_{1}} & 0\\ 0 & \tilde{B_{2}} \end{array}\right], \end{array}$$
  (32) $$\begin{array}{cc} svd\left(\tilde{B_{i}}\right) & =svd\left(\tilde{B_{1}}\right)+svd\left(\tilde{B_{2}}\right), \end{array}$$
  where $svd\left(\tilde{B_{i}}\right)$ is the singular value decomposition of the matrix $\tilde{B_{i}}$; in this case, we compute the matrix $\tilde{B_{2}}$ first. If the split occurs at *i* equal to *n*, then the matrix $\tilde{B_{2}}$ is equal to $q_{n}$ and $q_{n}$ is a singular value.
**Cancellation:**  If the matrix entry $q_{i}$ is equal to zero, we split the matrix $\tilde{B_{i}}$ by using Givens rotations from the left to zero out row *i* as follows:
  (33) $$\begin{array}{c} G_{i,i+1}^{T}\left[\begin{array}{ccccccc} q_{1} & e_{2} & \; & & \; & & \\ \; & \ddots & \ddots & \; & & \; & \\ \; & \; & q_{i-1} & e_{i} & \; & & \\ \; & \; & & 0 & e_{i+1} & \; & \\ \; & \; & & \; & q_{i+1} & \ddots & \\ \; & \; & & \; & & \ddots & e_{n}\\ \; & \; & & \; & & \; & q_{n} \end{array}\right]=\\ \left[\begin{array}{ccccccc} q_{1} & e_{2} & \; & & \; & & \\ \; & \ddots & \ddots & \; & & \; & \\ \; & \; & q_{i-1} & e_{i} & \; & & \\ \; & \; & & 0 & 0 & b & \\ \; & \; & & \; & q_{i+1} & \ddots & \\ \; & \; & & \; & & \ddots & e_{n}\\ \; & \; & & \; & & \; & q_{n} \end{array}\right], \end{array}$$
  whereas the budge *b* is removed by using the Givens rotations for k=i+2,…,n. Since the matrix element $e_{i+1}$ is equal to zero, the matrix splits again into two block diagonal sub-matrices (see the splitting step).
**Negligibility:**  The values of the matrix elements $e_{i}$ or $q_{i}$ will be small but not exactly zero due to the finite precision arithmetic used by digital processors. Therefore, we require a threshold to decide when the elements $e_{i}$ or $q_{i}$ can be considered zero. Golub and Reinsch [58] recommend the following threshold rule:
  (34) $$|e_{i+1}\left|,\right|q_{i}|\leq \varepsilon \max_{i}\left(|q_{i}\left|+\right|e_{i}|\right)=\varepsilon B_{1},$$
  where ε is the machine precision. Björck [27] suggests the following approach:
  (35) $$\begin{array}{cc} |e_{i+1}| & \leq 0.5\varepsilon \left(|q_{i}\left|+\right|q_{i+1}|\right), \end{array}$$
  (36) $$\begin{array}{cc} |q_{i+1}| & \leq 0.5\varepsilon \left(|e_{i}\left|+\right|e_{i+1}|\right). \end{array}$$
  Linpack [5] uses a variant Björck’s approach that omits the factor 0.5 in Equations (35) and (36).

## 5. Usage of the RcdMathLib

RcdMathLib can be used on PCs, resource-constrained devices such as microcontrollers, or on small single-board computers like Raspberry Pi devices. It is a free software and available under the terms of the GNU Lesser General Public License as published by the Free Software Foundation, version 2.1 (LGPLv2.1) [59]. The RcdMathLib software is written in the C programming language by using the GNU Compiler Collection (GCC) for full-fledged devices and embedded tool chains for resource-limited devices; for instance, the GNU ARM Embedded Toolchain. RcdMathLib can also be used on top of an Operating System (OS) for resource-constrained devices with a minimal effort due to the modular architecture of the library. We support the RIOT-OS, which is an open source IoT OS [60]. RcdMathLib is interfaced with the RIOT-OS using the GNU Make utility, whereby the user only needs to choose the modules needed by setting the USE_MODULE-macro. We automatically calculate the dependencies of the modules needed and the user can choose between a floating-point single-precision or double-precision depending on the available stack memory. An OS for resource-limited devices is recommended for the use of the RcdMathLib, but it is not required. A minimum stack size of 2560 bytes is needed to compute with floating-point numbers. The printf() function needs extra memory stack, therefore a minimum stack size of 4608 bytes is required to work with double-precision for floating-point arithmetic. We recommend a stack size of 8192 bytes.

The Linaro toolchain can be used on Linux or Windows to build applications for a target platform [61]. The OpenOCD can be used for flashing the code to the target (chip) as well as for low level or source level debugging [62]. The source code as well as the documentation (APIs) of the RcdMathLib can be downloaded from the GitLab repository [63]. The wikis are available on the homepage of the library to get started with the RcdMathLib [9].

### Simple Example

We present a simple example to demonstrate how to use the RcdMathLib by defining two $\left(3,4\right)$ matrices in Listing 1. We create a matrix with specific values equal to π in the main diagonal by calling the “matrix_get_diag_mat()” function. In the next step, we calculate the transpose of the second matrix by invoking the “matrix_get_transpose()” function. Finally, we calculate the multiplication of the first matrix with the transpose of the second matrix by executing the “matrix_mul()” function. In these three cases, the user should deliver the dimension of the matrices as well as a reference to the matrix resulted. The outputs of the calculated results are presented in Listing 2.

**Listing 1:**
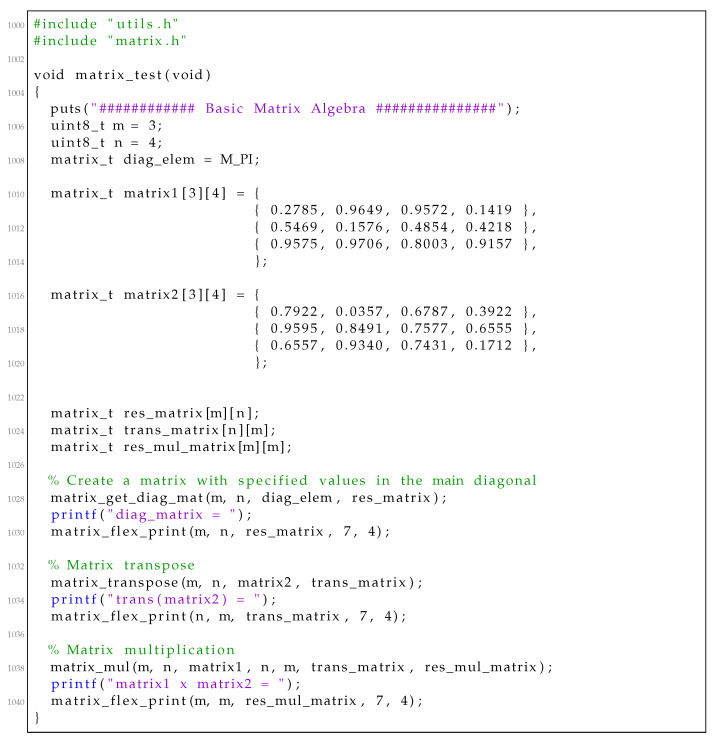
Example of basic matrix algebra.

**Listing 2:**
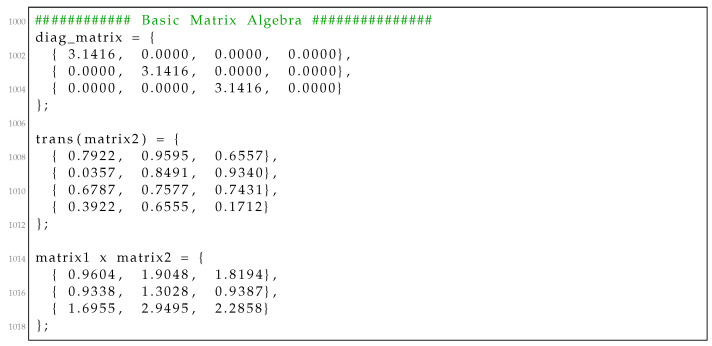
Outputs of the example of basic matrix algebra.

## 6. Evaluation of the Algorithms

We evaluated the linear as well as the nonlinear algebra modules on an STM32F407 Microcontroller Unit (MCU) based on the ARM Cortex-M4 core operating at 168 MHz and having a memory capacity of 192 KB RAM. In order to demonstrate the scalability of the algorithms implemented, we also evaluated the same algorithms on Raspberry Pi 3, which has more capacity (Quad Core 1.2 GHz and 1 GB RAM) than the STM32F4-MCU.

### 6.1. Evaluation of the Linear Algebra Module

We evaluated the linear algebra module by using a (m×n) matrix *A* and a vector $\vec{b}$ with uniformly distributed random numbers. The aim is to calculate and measure the mean execution time of the methods for solving linear equation systems. We evaluated the SVD-, QR-, and the LU-based algorithms for solving linear equation systems described in Section 3.1. The determined and the over-determined linear equation systems can be represented by the colon notation as follows:

(37) $$A\left(1:i,1:i\right)\vec{x}=\vec{b}\left(1:i\right),$$

where 2≤i≤n, and

(38) $$A\left(1:i,1:n\right)\vec{x}=\vec{b}\left(1:i\right),$$

where n+1≤i≤m and A(1:i,1:n) is the sub-matrix of A with rows 1 up to *i* and columns 1 up to *n*. We use the same format as the corresponding column notation in MATLAB. The determined and the over-determined linear equation systems are illustrated in the matrix form in Equations (39) and (40), respectively.

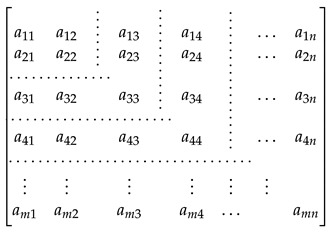
(38)

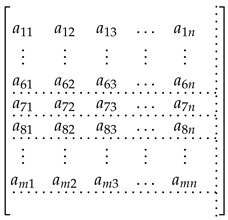
(39)

The horizontal and vertical dotted lines enclose the square and rectangular sub-matrices in Equations (39) and (40). We set the maximal row (*m*) and column (*n*) numbers to 10 and 5. We also measured the mean execution time of the matrix multiplication by using the same matrix $A_{m,n}$ and a matrix $B_{\left(n,p\right)}$ initialized with uniformly distributed random numbers. The row and column number of the matrix *B* are equal to 5 and 10. The row number (*m*) of the matrix *A* varies from 1 to 10. The linear equation systems are solved by using three different decomposition algorithms: the Golub–Kahan–Reinsch, Householder, and GE with pivoting. We measured the execution time of matrix multiplications as well as of the methods for solving linear equation systems on the STM32F4 MCU and the Raspberry Pi 3.

**Figure 4:**
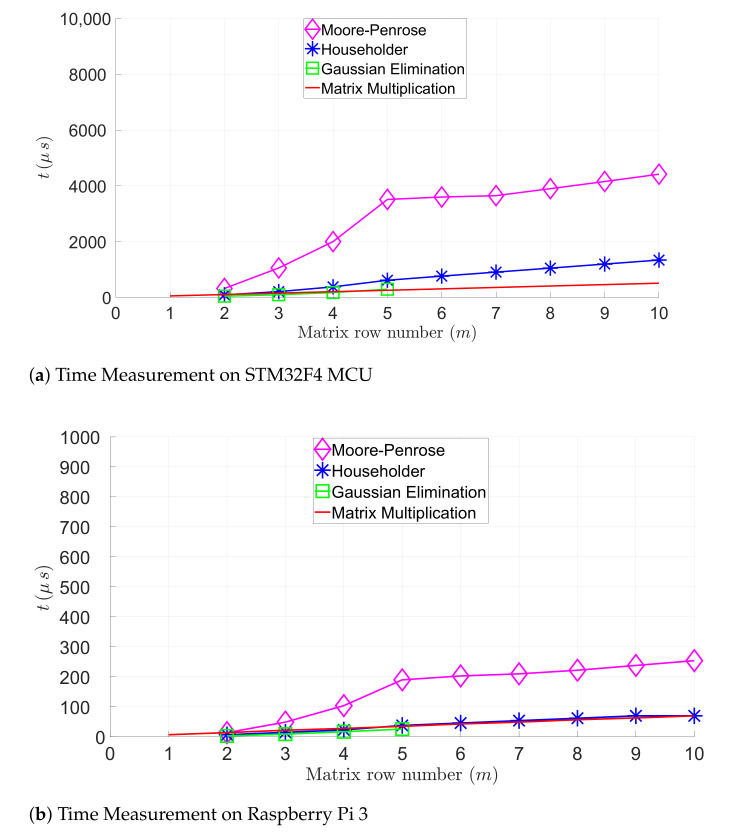
Computing time evaluation of matrix multiplications and linear equation systems.

For solving linear equation systems, Figure 4 compares the execution time of the following algorithms: GE with pivoting, the Householder, and the Golub–Kahan–Reinsch. Furthermore, Figure 4 represents the execution time of the matrix multiplications. Figure 4a,b illustrate the execution time of these algorithms in function of the row number of the matrix on the STM32F4 MCU and the Raspberry Pi 3. The Golub–Kahan–Reinsch-based algorithm has the largest execution time, since it is more expensive than other algorithms (see Table 2). Table 3 summarizes the mean execution time of the matrix evaluated by calculating an $A_{10,5}\times B_{5,7}$ matrix or solving an $\left(7,5\right)$ linear equation system. These execution times are measured on the STM32F4 MCU and the Raspberry Pi 3. The Raspberry Pi 3 outperforms the STM32F4 MCU, as expected, due to the limited computing capacity of the STM32F4 MCU. However, the execution time for finding a solution applying the Golub–Kahan–Reinsch algorithm remains in a micro-second range on the STM32F4 MCU, which would be sufficient for many IoT applications.

### 6.2. Evaluation of the Non-Linear Algebra Module

The nonlinear algebra module is evaluated by using exponential and sinusoidal data [64]. Optimizing of least-squares problems are solved by using the modified GN and LVM methods as described in Section 3.2.1 and Section 3.2.2.

#### 6.2.1. Evaluation with Exponential Data

Given the model function $g(\vec{x},t)$ that is equal to:

(41) $$g\left(\vec{x},t\right)=x_{1}e^{x_{2}t},$$

where $\vec{x}={\left[x_{1},x_{2}\right]}^{T}$ and $\vec{x_{0}}=\left[6,0.3\right]$ is the initial guess. The data set is $d(t_{i},y_{i})$, whereby $t_{i}$ is equal to {1,…,8} and $y_{i}$ is equal to {8.3,11.0,14.7,19.7,26.7,35.2,44.4,55.9}.

The aim is to find the parameters ($x_{1},x_{2}$) that most accurately match the model function $g(\vec{x},t)$ by minimizing the sum of squares of the error function $f_{i}$. The function $f_{i}$ computes the residual values and is equal to:

(42) $$\begin{array}{cc} f_{i}\left(x_{1},x_{2}\right) & =x_{1}e^{x_{2}t_{i}}-y_{i}. \end{array}$$

We introduce the error function vector $\vec{f}$:

(43) $$\begin{array}{cc} \vec{f}\left(x_{1},x_{2}\right) & ={\left[\begin{array}{c} x_{1}e^{x_{2}}-y_{1},\;\ldots ,x_{1}e^{8x_{2}}-y_{8} \end{array}\right]}^{T}. \end{array}$$

The Jacobian matrix is equal to:

(44) $$J_{f}=\left[\begin{array}{ccc} \frac{\partial f_{1}}{\partial x1} & \frac{\partial f_{1}}{\partial x_{2}}\\ \frac{\partial f_{2}}{\partial x1} & \frac{\partial f_{2}}{\partial x_{2}}\\ \vdots & \vdots & \\ \frac{\partial f_{n}}{\partial x1} & \frac{\partial f_{n}}{\partial x_{2}} \end{array}\right]=\left[\begin{array}{ccc} e^{x_{2}} & e^{x_{2}}x_{1}\\ e^{2x_{2}} & 2e^{2x_{2}}x_{1}\\ \vdots & \vdots & \\ e^{8x_{2}} & 8e^{8x_{2}}x_{1} \end{array}\right]$$

**Figure 5:**
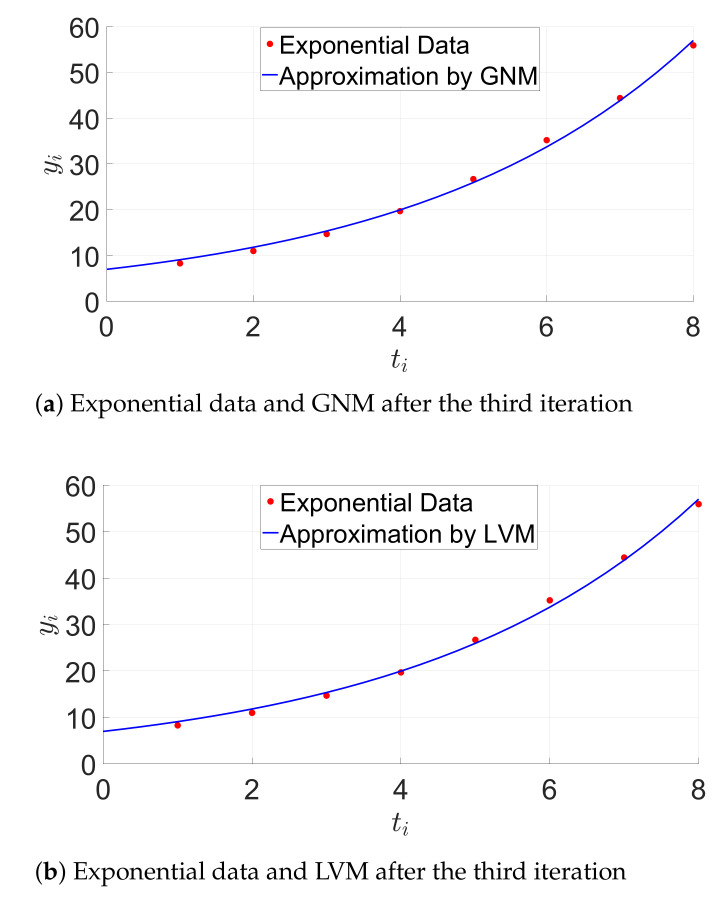
Exponential model approximation. GNM, Gauss–Newton Method. LVM, Levenberg–Marquardt Method.

The initial square residual $∥\vec{f}\left(\vec{x_{0}}\right){∥}_{2}^{2}$ is equal to 127.309. We get the solution $\vec{x_{3}}={\left[7.000093,0.262078\right]}^{T}$ by using the GN algorithm after three iterations. The appropriate square residual $∥\vec{f}\left(\vec{x_{3}}\right){∥}_{2}^{2}$ is equal to 6.013, which indicates the improvement of the model. We obtain the solution $\vec{x_{3}}={\left[7.000090,0.262078\right]}^{T}$ by using the LVM algorithm after three iterations. The LVM algorithm shows nearly the same behavior as the modified GN method. This is confirmed by Figure 5.

Table 4 summarizes the average time per iteration required from the GNM and LVM algorithms on the STM32F4 MCU and the Raspberry Pi 3.

#### 6.2.2. Evaluation with Sinusoidal Data

The model function is:

(45) $$g\left(\vec{x},t\right)=x_{1}\sin\left(x_{2}t+x_{3}\right)+x_{4},$$

whereby, $\vec{x}={\left[x_{1},x_{2},x_{3},x_{4}\right]}^{T}$ and $\vec{x_{0}}=\left[17,0.5,10.5,77\right]$ is the initial guess. The set of data points is $d(t_{i},y_{i})$, where $t_{i}$ is equal to {1,…,12} and $y_{i}$ is equal to {61,65,72,78,85,90,92,92,88,81,72,63}. The error function is $f_{i}=x_{1}\sin\left(x_{2}t_{i}+x_{3}\right)+x_{4}-y_{i}$; therefore, the error function vector $\vec{f}$ is:

(46) $$\begin{array}{cc} \vec{f}\left(x_{1},x_{2},x_{3},x_{4}\right) & =\left[\begin{array}{c} x_{1}\sin\left(x_{2}+x_{3}\right)+x_{4}-y_{1}\\ x_{1}\sin\left(2x_{2}+x_{3}\right)+x_{4}-y_{2}\\ \vdots \\ x_{1}\sin\left(12x_{2}+x_{3}\right)+x_{4}-y_{12} \end{array}\right]. \end{array}$$

Thus, the Jacobian matrix is calculated using the partial derivatives in Equation (44) and is equal to:

(47) $$J_{f}=\left[\begin{array}{ccc} \sin\left(x_{2}+x_{3}\right) & x_{1}\cos\left(x_{2}+x_{3}\right) & x_{1}\cos\left(x_{2}+x_{3}\right)\\ \sin\left(2x_{2}+x_{3}\right) & 2x_{1}\cos\left(2x_{2}+x_{3}\right) & x_{1}\cos\left(2x_{2}+x_{3}\right)\\ \vdots & \vdots & \vdots \\ \sin\left(12x_{2}+x_{3}\right) & 12x_{1}\cos\left(12x_{2}+x_{3}\right) & x_{1}\cos\left(12x_{2}+x_{3}\right) \end{array}\right]$$

**Figure 6:**
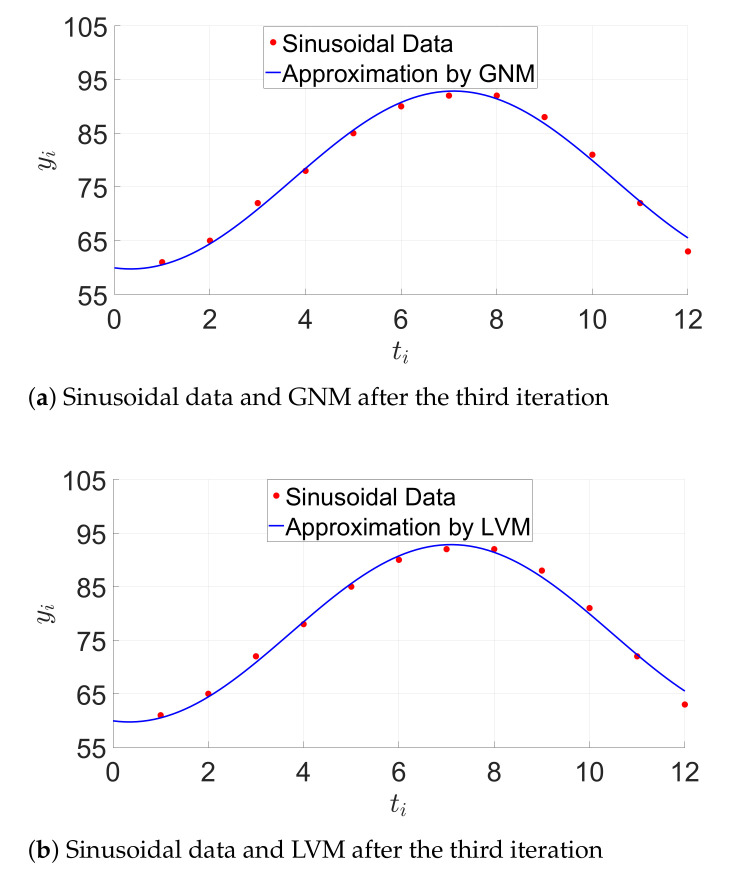
Sinusoidal model approximation. GNM, Gauss–Newton Method. LVM, Levenberg–Marquardt Method.

The initial square residual $∥\vec{f}\left(\vec{x_{0}}\right){∥}_{2}^{2}$ is equal to 40.048. After one iteration ($∥\vec{f}\left(\vec{x_{0}}\right){∥}_{2}^{2}=13.805$), the LVM algorithm is slightly more efficient than the GN method ($∥\vec{f}\left(\vec{x_{0}}\right){∥}_{2}^{2}=13.810$). Both algorithms show the same behavior after two iterations. Figure 6 shows the sinusoidal model after three iterations by using the GN and LVM algorithms.

Table 5 summarizes the average time per iteration required from the GNM and LVM approaches on the STM32F4 MCU and the Raspberry Pi 3.

## 7. Conclusions and Outlook

We presented an open source library for linear and nonlinear algebra as well as an application module for localization that is suitable for resource-limited, mobile, and embedded systems. This library permits the solution of linear equations and matrix operations like the matrix decomposition, the calculation of the inverse, or the rank of a matrix. It provides various algorithms such as sorting or filtering algorithms. This software also enables solving nonlinear problems like curve fitting or nonlinear equations. RcdMathLib allows for the localization of mobile devices by using localization algorithms like, for instance, the trilateration. The localization can be further refined by using an adaptive optimization algorithm based on the SVD method. The localization software module facilitates the localization of the mobile device in NLoS scenarios by using a multipath distance detection and mitigation algorithm. RcdMathLib can serve as a basis for artificial intelligence techniques for mobile technologies with IoT, or as a tool in research and industry. Therefore, we intend to extend the RcdMathLib with machine learning or digital signal processing algorithms. We also aim to extend the package with an additional algorithm for solving nonlinear problems called the Landweber method [65].

## Figures and Tables

**Table 1:** Comparison of mathematical libraries. FFT, Fast Fourier Transform.

Library	Capabilities	Platform
GNU Scientific Library (GSL)	Basic linear algebra, FFT, basic mathematical routines	No support for resource-limited devices
C standard mathematical library <math.h>	Basic mathematical functions	Support for resource-limited devices
IQmath and Qmath	Basic mathematical functions	MSP430 and MSP432 microcontrollers
Libfixmatrix	Basic mathematical functions and matrix inversion	ARM Cortex-M3 processors
MicroBLAS	Basic linear algebra	PC and microcontrollers
MatrixMath and BasicLinearAlgebra	Basic matrix operations	Restricted to the Arduino platform
NumPy, SciPy	Linear algebra computational mathematics	No support for resource-limited devices
math and cmath (MicroPython)	Basic mathematical functions	ARM Cortex-M processors or CircuitPython- powered boards
*RcdMathLab*	*mathematical* *functions for* *nonlinear,* *linear algebra* *optimization,* *and localization.* *Utilities*	*PCs and* *microcontrollers:* *Platforms using* *C compiler*

**Table 2:** Complexity of the algorithms evaluated.

Algorithm	Complexity [Flops]
Matrix multiplication: $A_{m,n}\times B_{n,p}$	mp(2n−1)
QR-Householder	$2mn^{2}-\frac{2}{3}n^{3}$
Golub–Kahan–Reinsch	$4m^{2}n+8mn^{2}+9n^{3}$

**Table 3:** Mean execution time of computing $A_{7,5}\times B_{5,10}$ or solving an $\left(7,5\right)$ linear equation system.

Algorithm	Mean Execution Time (s)	
	STM32F4	Raspberry Pi 3
Matrix multiplication: $A_{7,5}\times B_{5,10}$	356	49
Householder-based solution	906	54
Golub–Kahan–Reinsch-based solution	3647	204

**Table 4:** Mean execution time of the Gauss–Newton and Levenberg–Marquardt methods (per iteration) using exponential data.

Algorithm	Mean Execution Time (s)	
	STM32F4	Raspberry Pi 3
Gauss–Newton	1065	35
Levenberg–Marquardt	1194	42

**Table 5:** Mean execution time of the Gauss–Newton and Levenberg–Marquardt methods (per iteration) using sinusoidal data.

Algorithm	Mean Execution Time (s)	
	STM32F4	Raspberry Pi 3
Gauss–Newton	3165	157
Levenberg–Marquardt	2825	141

## Data Availability

Source code can be download from the GitLab repository on Reference [63].
